# Platelet Transfusion and Death or Neurodevelopmental Impairment in Children Born Extremely Preterm

**DOI:** 10.1001/jamanetworkopen.2023.52394

**Published:** 2024-01-23

**Authors:** Patricia E. Davenport, Thomas R. Wood, Patrick J. Heagerty, Martha C. Sola-Visner, Sandra E. Juul, Ravi M. Patel

**Affiliations:** 1 Division of Newborn Medicine, Boston Childrens Hospital, Boston, Massachusetts; 2 Division of Neonatology, University of Washington, Seattle; 3 Institute on Human Development and Disability, University of Washington, Seattle; 4 Department of Biostatistics, University of Washington, Seattle; 5 Department of Pediatrics, Emory University School of Medicine and Childrens Healthcare of Atlanta, Atlanta, Georgia

## Abstract

**Importance:**

Infants born extremely preterm receive transfusions at higher platelet count thresholds than older children and adults due to concerns for intracranial hemorrhage. A recent randomized trial comparing 2 platelet transfusion thresholds showed the higher threshold was associated with increased risk of long-term adverse neurodevelopmental outcomes.

**Objective:**

To evaluate the association of platelet transfusion exposure with death and severe neurodevelopmental impairment (NDI) at 2 years’ corrected age in a cohort of infants born extremely preterm.

**Design, Setting, and Participants:**

An observational cohort study and secondary analysis of the Preterm Erythropoietin Neuroprotection Trial, a randomized, placebo-controlled clinical trial of erythropoietin neuroprotection in neonates born extremely preterm, was conducted in 30 neonatal intensive care units in the US from December 1, 2013, to September 31, 2016. This analysis included 819 infants born extremely preterm at 24 to 27 completed weeks of gestation who had a documented outcome (death or neurodevelopmental assessment). Analysis was performed in April 2023.

**Exposures:**

Any platelet transfusion during neonatal intensive care unit hospitalization.

**Main Outcomes and Measures:**

The primary composite outcome was death or severe NDI evaluated at 2 years’ corrected age using the Bayley Scales of Infant Development–Third Edition (BSID-III) and the Gross Motor Function Classification System and was defined as the presence of severe cerebral palsy or a BSID-III composite motor or cognitive score 2 SDs below the mean. Confounding by indication for platelet transfusion was addressed with covariate adjustment and propensity score methods.

**Results:**

Of the 819 infants included in the analysis (429 [52.4%] male; mean [SD] gestational age, 25.5 [1.1] weeks), 245 (30.0%) received at least 1 platelet transfusion during their initial hospitalization. The primary outcome occurred in 46.5% (114 of 245) of infants exposed to a platelet transfusion and 13.9% (80 of 574) of nonexposed infants with a corresponding odds ratio of 2.43 (95% CI, 1.24-4.76), adjusted for propensity score, gestational age at birth, and trial treatment group. The individual components of death and severe NDI were directionally consistent with the overall composite outcome.

**Conclusions and Relevance:**

The findings of this study suggest that platelet transfusion in infants born extremely preterm may be associated with an increased risk of death or severe NDI at 2 years’ corrected age, although the possibility of residual confounding by indication cannot be excluded.

## Key Points

**Question:**

Are platelet transfusions associated with death or neurodevelopmental impairment in children born extremely preterm?

**Findings:**

In this cohort study of 819 infants born extremely preterm enrolled in a clinical trial of erythropoietin neuroprotection, infants exposed to platelet transfusion had a statistically significant higher incidence of death or severe neurodevelopmental impairment at 2 years’ corrected age compared with nonexposed infants (46.5% vs 13.9%). In separate analyses, death and severe NDI were directionally consistent with the overall composite outcome.

**Meaning:**

The findings of this study suggest that infants born extremely preterm who receive platelet transfusions may have a higher risk of death or neurodevelopmental impairment.

## Teaser

This cohort study examines the presence of death or severe neurodevelopmental impairment at 2 years’ corrected age in infants born extremely preterm who received platelet transfusions.

## Introduction

Thrombocytopenia, defined as a platelet count less than 150 × 10^3^/μL (to convert to ×10^9^/L, multiply by 1), is a common neonatal problem that affects 22% to 35% of infants admitted to the neonatal intensive care unit (NICU).^1^ Platelets are the primary hemostatic cells in circulation and are crucial to prevent blood extravasation after vascular injury. Prophylactic platelet transfusions are routinely administered to preterm infants with nonbleeding thrombocytopenia in hopes of preventing bleeding.^2^ However, multiple studies have shown a lack of association between the platelet count and neonatal bleeding severity, suggesting that other factors aside from the platelet count are determinants of neonatal bleeding risk.^3,4,5,6^ Simultaneously, multiple retrospective and observational studies reported an association between neonatal platelet transfusions and increased morbidity and mortality,^7,8,9,10,11^ but without strong clinical data to guide transfusion decision-making, neonatal platelet transfusion practices continued to vary widely between clinicians, centers, and countries.^12,13,14^ In 2019, a multicenter randomized trial reported comparisons of a platelet transfusion threshold of 50 × 10^3^/μL (high threshold) to 25 × 10^3^/μL (low threshold) in 660 preterm infants born at less than 34 weeks’ gestation.^15^ Ninety percent of infants randomized to the high threshold group received at least 1 platelet transfusion, compared with 53% of infants randomized to the low platelet transfusion threshold. In analysis of the primary outcome, the study found a higher incidence of death and/or major bleeding in infants randomized to the high compared with the low platelet transfusion threshold arm. A 2-year neurodevelopmental follow-up from this trial was recently published.^16^ Follow-up data were available for 601 (92%) of eligible participants, with 50% of children previously randomized to the high threshold group experiencing death or survival with unfavorable neurodevelopmental outcome, compared with 39% in the low threshold group (odds ratio [OR], 1.54; 95% CI, 1.09-2.17; *P* = .02). Comparing high vs low threshold groups, the incidence of death at 2 years was 30% vs 24% (OR, 1.36; 95% CI, 0.93-1.99) and unfavorable neurodevelopmental outcome among survivors was 27% vs 20% (OR, 1.53; 95% CI, 0.95-2.45). However, formal neurocognitive testing was not performed in all participants, so the ability to determine the association between platelet transfusions and specific aspects of neurodevelopment (cognitive, motor, and language) was limited. Additionally, the outcome of platelet dose, such as number of transfusions, at age 2 years is uncertain.

In this cohort study, we performed an observational, secondary analysis of data from the Preterm Erythropoietin Neuroprotection Trial (PENUT) trial^17,18^ to investigate the association between platelet transfusions administered to infants born extremely preterm and neurodevelopmental outcome at 2 years’ corrected age determined by assessments using the Bayley Scales of Infant and Toddler Development-III (BSID-III).

## Methods

### Data Source and Study Population

All infants enrolled in the PENUT trial were eligible for this study. The PENUT trial was a randomized placebo-controlled clinical trial of erythropoietin neuroprotection in neonates born extremely preterm conducted in 30 NICUs in the US from December 1, 2013, to September 31, 2016. Original trial exclusion criteria included life-threatening anomalies, chromosomal anomalies, disseminated intravascular coagulopathy, twin-to-twin transfusion, a hematocrit level above 65% (to convert to proportion of 1.0, multiply by 0.01), hydrops fetalis, or known congenital infection. Of the 941 infants enrolled in the PENUT trial, we included the 819 who had a documented outcome (death or neurodevelopmental assessment at 2 years’ corrected age). The PENUT trial was approved by institutional review boards at each site. Parental informed consent was obtained before or after birth. This study followed the Strengthening the Reporting of Observational Studies in Epidemiology (STROBE) reporting guideline.

Data were collected regarding maternal, pregnancy, delivery, and infant characteristics. Maternal race was collected as self-reported race that was obtained during consenting and enrollment. No imputation was performed for missing data. For this analysis, the study population was divided based on the primary exposure of platelet transfusion into infants who received any platelet transfusion during their NICU admission and those who did not receive a platelet transfusion. The total number of platelet transfusions received by each infant during admission was also recorded.

### Primary Outcome

The primary outcome was death or severe neurodevelopmental impairment (NDI) at approximately 2 years’ (ranging from 22 to 26 months) corrected age. Infants underwent evaluation using the BSID-III, administered by a certified examiner to assess cognitive, motor, and language development, and the Gross Motor Function Classification System (GMFCS) to evaluate for cerebral palsy. Severe NDI was defined as the presence of severe cerebral palsy (GMFCS score >2) or a BSID-III composite motor score or composite cognitive score 2 SDs below the mean (<70).

### Secondary Outcomes

Secondary outcomes included the composite of death or moderate to severe NDI (moderate NDI was defined as a GMFCS level of 2 or a BSID-III composite motor or cognitive score 1 SD below the mean [<85]) and the individual BSID-III cognitive, motor, and language scores.

### Multivariable Modeling and Propensity Score Methods

In considering confounding variables, we focused on covariates that potentially impact exposure to platelet transfusions and/or influence bleeding risk,^19^ which were measured before platelet transfusion. Based on this, we selected the following variables for consideration of inclusion in propensity score models: birth weight, small for gestational age (SGA) or intrauterine growth restriction, mechanical ventilation at enrollment, and the development of necrotizing enterocolitis or late-onset sepsis. Additional variables were considered after evaluating exposure groups, as described in the next section, as was recruitment site. We did not consider any variables or outcomes that occurred following 1 or more platelet transfusions to avoid adjusting for intermediate morbidities that might explain the association between platelet transfusion and our primary outcome.

To examine the outcome of platelet exposure, we generated stabilized inverse probability treatment weights (IPTWs) from a propensity score for any platelet exposure.^20,21^ Baseline variables, including morbidities before platelet transfusion as described in the previous paragraph, along with additional variables that differed between exposure groups with a *P* value <.10, were selected to include in the propensity score model. The following variables were included: maternal hypertension, premature labor, prolonged rupture of membranes, chorioamnionitis, cesarean delivery, prenatal antibiotics, gestational age, birth weight (because birth weight was included in addition to gestational age, SGA or intrauterine growth restriction was not additionally included), intubation and/or chest compressions during resuscitation, 5-minute Apgar score less than 5, sick appearance at birth, mechanical ventilation at enrollment, baseline platelet count, baseline hematocrit level, and preplatelet transfusion sepsis, necrotizing enterocolitis, or spontaneous intestinal perforation. In addition, due to practice variation by site, site was included as a variable in development of the propensity score. Standardized mean differences (SMDs) were used to estimate differences across exposure groups after weighting (eFigure 1 in the Supplement). Common support assumptions were evaluated by comparing propensity scores (values and quintiles) by platelet exposed and unexposed groups (eFigure 2 in the Supplement). For the primary analysis, the propensity score was specified as a covariate in the multivariable model as described in detail below.

In our main analyses, we chose to include data from all infants (n = 574 unexposed, n = 245 exposed) rather than excluding infants by matching by propensity score. Inverse probability treatment weights were used to perform weighted generalized estimating equation (GEE) regression examining the association between platelet exposure and outcome after adjustment for gestational age, trial treatment arm, and correlation between siblings, as described in the Statistical Analysis section. When examining platelet exposure as a continuous variable (number of exposures), proposed methods to develop stabilized IPTWs for continuous exposures did not adequately balance group differences across confounders^22,23^; therefore, the binary exposure IPTW was used in these weighted GEE models. As the propensity score for platelet exposure was also correlated with number of platelet exposures, continuous models with number of platelet transfusions as the exposure included additional adjustment for propensity score, modeled as a linear spline with knots at the 20th, 40th, 60th, and 80th percentiles. In addition to the primary analyses, we performed analyses to estimate the odds of the individual components of the primary outcome (death, severe NDI) as well as moderate or severe NDI.

### Sensitivity Analyses

We conducted 5 sensitivity analyses to address different assumptions in models of platelets as a binary exposure and 4 sensitivity analyses with number of platelet transfusions as a continuous exposure. These additional analyses included different propensity score methods (eTable 1 in the Supplement).^21^ As assessment of SMD between exposure groups revealed some ongoing imbalances in certain confounders (SMD >0.1), analysis 1 included baseline platelet count and hematocrit level, premature rupture of membranes, and mechanical ventilation at enrollment as additional separate covariates in the main model. Rather than using weighted regression, analysis 2 (binary exposure only) instead stratified by quintile of propensity score by including quintile dummy variables as covariates in the model. Analysis 3 was performed by 1:1 matching nearest propensity scores between exposure groups using the MatchIt library in R, version 4.3.1 (R Foundation for Statistical Computing). This resulted in 245 participants in each group being compared, with a GEE model accounting for clustering by matched pair. Analysis 4 used weighted GEE after optimal trimming of outlying propensity scores (n = 307 unexposed, n = 104 exposed).^24^ Analysis 5 involved full multivariable regression models with all propensity score variables included as separate covariates.

### Statistical Analysis

Data analysis for the present study was performed in April 2023. We report descriptive statistics for exposure groups (platelet transfusion vs no platelet transfusion) to describe the demographic and baseline maternal and infant characteristics. We expected infants who received platelet transfusions to be sicker than those unexposed, so we addressed confounding by indication for platelet transfusion using propensity score modeling approaches, as described in the Multivariable Modeling and Propensity Score section. Exposed and unexposed infants were compared using a Wald test after linear or logistic GEEs regression models with robust SEs adjusting for gestational age and erythropoietin treatment arm in the PENUT trial, and clustering structure to account for potential correlation of outcomes for same-birth siblings.^25^ Statistical significance was considered a 2-sided *P* <.05. All analyses were conducted with R, version 4.3.1, software (R Foundation).

## Results

### Cohort Characteristics

From the original cohort of 941 infants enrolled in the PENUT trial, 122 were lost to follow-up and had missing primary outcome data, resulting in a total of 819 infants included in this secondary analysis (eFigure 3 in the Supplement). Of these, 692 were assessed in at least 1 BSID-III subscale at follow-up and the remainder either died (n = 113) or were alive at follow-up with known primary outcome but not formally assessed (n = 14). The study population included 429 males (52.4%) and 390 females (47.6%), with a mean (SD) gestational age of 25.5 (1.1) weeks and birth weight of 798 (191) grams. Of the included infants, 245 (30.0%) received at least 1 platelet transfusion during NICU admission. Platelet transfusion rates by study center are shown in eFigure 4 in the Supplement. Infants who received platelet transfusions had a lower gestational age, birth weight, 5-minute Apgar score, and baseline platelet counts and a higher incidence of being SGA, requiring intubation and/or chest compressions at birth, sick appearance at birth, requiring mechanical ventilation at the time of enrollment, severe sepsis, necrotizing enterocolitis, spontaneous intestinal perforation, and severe intraventricular hemorrhage (Table 1). Regarding differences in maternal, pregnancy, or delivery characteristics between the cohorts, infants who received a platelet transfusion had a higher incidence of maternal hypertension and need for cesarean delivery and a lower incidence of preterm labor, prolonged rupture of membranes, chorioamnionitis, and administration of prenatal antibiotics (Table 1).

**Table 1.** Baseline and Clinical Characteristics

Characteristic	No. (%)		*P* value
	No platelet transfusion (n = 574)	≥1 Platelet transfusions (n = 245)	
**Maternal factors**			
Race^a^			
Black	121 (21.1)	67 (27.3)	.28
White	392 (68.3)	161 (65.7)	
Other	61 (10.6)	17 (6.9)	
Education			
High school or less	184 (32.1)	73 (29.8)	.23
Some college	183 (31.9)	67 (27.3)	
College degree or greater	145 (25.3)	71 (29.0)	
Unknown or not reported	62 (10.8)	34 (13.9)	
Obesity	53 (9.2)	31 (12.7)	.15
Gestational diabetes	32 (5.6)	12 (4.9)	.95
Hypertension	82 (14.3)	91 (37.1)	<.001
Received prenatal care	547 (95.3)	238 (97.1)	.38
Multiple gestation pregnancy	153 (26.7)	66 (26.9)	.92
Preterm labor	391 (68.1)	111 (45.3)	<.001
Prolonged rupture of membranes	178 (31.0)	46 (18.8)	<.001
Chorioamnionitis	85 (14.8)	22 (9.0)	.007
Prenatal antibiotics	215 (37.5)	77 (31.4)	.038
≥2 doses of antenatal corticosteroids	398 (69.3)	176 (71.8)	.55
Prenatal magnesium	455 (79.3)	202 (82.4)	.27
Cesarean delivery	372 (64.8)	194 (79.2)	<.001
**Infant factors**			
Gestational age, mean (SD), wk	25.7 (1.1)	25.1 (1.1)	<.001
Birth weight, mean (SD), g	846 (178)	684 (173)	<.001
Sex			
Female	277 (48.3)	113 (46.1)	.63
Male	297 (51.7)	132 (53.9)	
Small for gestational age	59 (10.3)	72 (29.4)	<.001
Intubation and/or chest compressions at birth	442 (77.0)	228 (93.1)	<.001
Apgar <5 at 5 min	85 (14.8)	85 (34.7)	<.001
Sick appearance at birth	226 (39.4)	130 (53.1)	.002
Mechanical ventilation at enrollment	435 (75.8)	233 (95.1)	<.001
Initial platelet count after birth, mean (SD), ×10^3^/μL	222 (69)	158 (72)	<.001
Initial hematocrit after birth, mean (SD), %	43.2 (6.1)	41.7 (7.5)	.008
Erythropoietin treatment	285 (49.7)	125 (51.0)	.44
Serious morbidities (at any point in hospital course)			
Severe sepsis	30 (5.2)	42 (17.1)	<.001
Necrotizing enterocolitis	18 (3.1)	46 (18.8)	<.001
Spontaneous intestinal perforation	10 (1.7)	21 (8.6)	<.001
Severe intraventricular hemorrhage	49 (8.5)	63 (25.7)	<.001

SI conversion factors: to convert hematocrit to proportion of 1.0, multiply by 0.01; platelets to ×10^9^/L, multiply by 1.

^a^ Data on race were included to describe the cohort, for consistency with other reports of the trial. Other included individuals with race and ethnicity other than Black or White.

### Primary Outcome in Infants With and Without Transfusion

**Figure 1.**
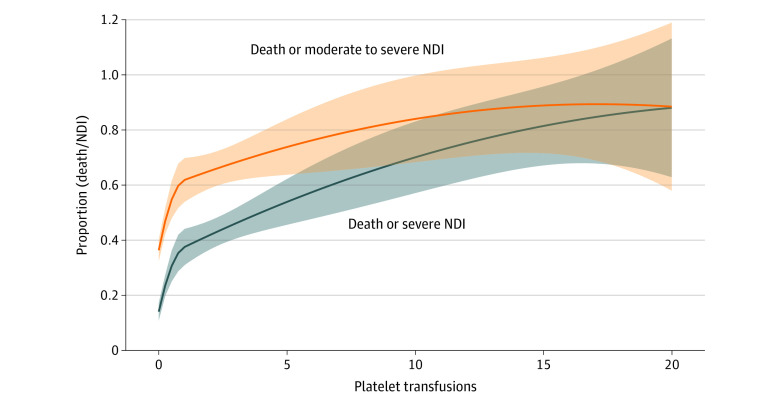
Probability of Death or Adverse Neurodevelopmental Outcomes by Number of Platelet Transfusions Probability of death or severe neurodevelopmental impairment (NDI) (primary outcome) and death or moderate to severe NDI are shown by the number of platelet transfusion exposures. Estimates were modeled as second-order polynomial splines with a knot at 1 platelet transfusion. Five infants who received more than 20 transfusions were assigned a value of 20. The shaded areas indicate 95% CIs.

The primary combined outcome of death or severe NDI occurred in 46.5% (114 of 245) of infants who received a platelet transfusion during admission and 13.9% (80 of 574) of those who did not receive a transfusion. After adjustment for propensity score, gestational age at birth, and treatment group, the adjusted OR (AOR) for death or severe NDI comparing platelet exposure to unexposed infants was 2.43 (95% CI, 1.24-4.76) (Table 2). The individual components of death and severe NDI were directionally consistent with the overall composite outcome, with 32.2% of the infants who received 1 or more platelet transfusion dying before 2 years’ corrected age compared with 5.9% in those who did not receive a transfusion with a corresponding AOR of 3.62 (95% CI, 1.64-8.02). Similarly, 21.9% of infants who received 1 or more platelet transfusion had severe NDI compared with 8.8% of those who did not receive any transfusion, with a corresponding AOR of 1.11 (95% CI, 0.52-2.36) (Table 2). Of the 245 infants exposed to platelet transfusion, 146 (59.6%) received more than 1, with a median (IQR, range) of 3 (IQR, 2-6; range, 2-46) platelet transfusion exposures. Analysis of the association between number of platelet transfusions and the primary outcome found that, per each additional platelet transfusion exposure, the AOR of death or severe NDI was 1.25 (95% CI, 1.07-1.45) (Table 2 and Figure 1).

**Table 2.** Primary and Secondary Outcomes

Outcome	No./total No. (%) or mean (SD)		AOR or AMD (95% CI)^a^	
	No platelet transfusion^b^	≥1 Platelet transfusions^b^	≥1 Platelet transfusion vs none^c^	Per each additional platelet transfusion^c^
**Primary outcome**				
Death or severe NDI	80/574 (13.9)	114/245 (46.5)	2.43 (1.24 to 4.76)	1.25 (1.07 to 1.45)
**Components of primary outcome**				
Death	34/574 (5.9)	79/245 (32.2)	3.62 (1.64 to 8.02)	1.22 (1.07 to 1.40)
Severe NDI (survivors)	46/521 (8.8)	35/160 (21.9)	1.11 (0.52 to 2.36)	1.14 (1.02 to 1.27)
**Secondary outcome**				
Death or moderate-severe NDI	207/574 (36.0)	166/245 (68.0)	1.62 (0.83 to 3.17)	1.15 (0.99 to 1.34)
Moderate-severe NDI (survivors)	173/523 (33.3)	87/161 (54.0)	1.02 (0.51 to 2.03)	1.06 (0.94 to 1.19)
**Continuous secondary outcome**				
BSID-III Cognitive (n=692)	92.3 (15.6)	87.1 (15.6)	3.2 (−4.3 to 10.8)	−0.2 (−1.4 to 0.9)
BSID-III Motor (n=680)	92.5 (16.0)	82.3 (17.2)	−4.7 (−8.5 to −0.9)	−1.1 (−2.2 to −0.01)
BSID-III Language (n=677)	89.6 (17.3)	82.9 (17.7)	−0.6 (−7.8 to 6.6)	−0.7 (−1.8 to 0.5)

Abbreviations: AMD, adjusted mean difference; AOR, adjusted odds ratio; NDI, neurodevelopmental impairment; BSID, Bayley Scales of Infant and Toddler Development.

^a^ Estimates are from model accounting for propensity score approaches as detailed in Methods and include additional adjustment for gestational age at birth and trial treatment arm. Continuous outcomes are additionally adjusted for maternal educational level.

^b^ Primary outcomes are reported as No./total No. (%) and continuous secondary outcomes are reported as mean (SD).

^c^ Primary outcomes are reported as AOR (95% CI) and continuous secondary outcomes are reported as AMD (95% CI).

### Secondary Outcomes in Infants With and Without Transfusion

**Figure 2.**
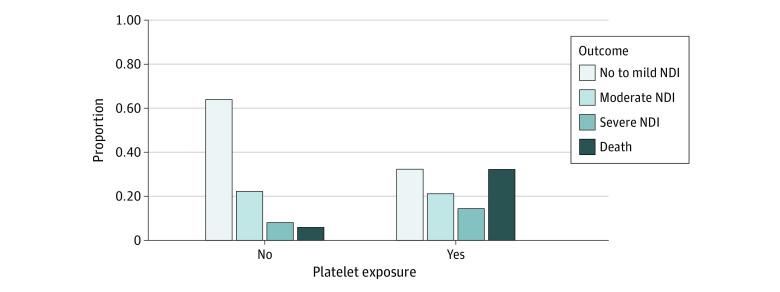
Distribution of Proportion of Infants with Death, Severe Neurodevelopmental Impairment (NDI), Moderate NDI, or No to Mild NDI by Platelet Exposure

**Figure 3.**
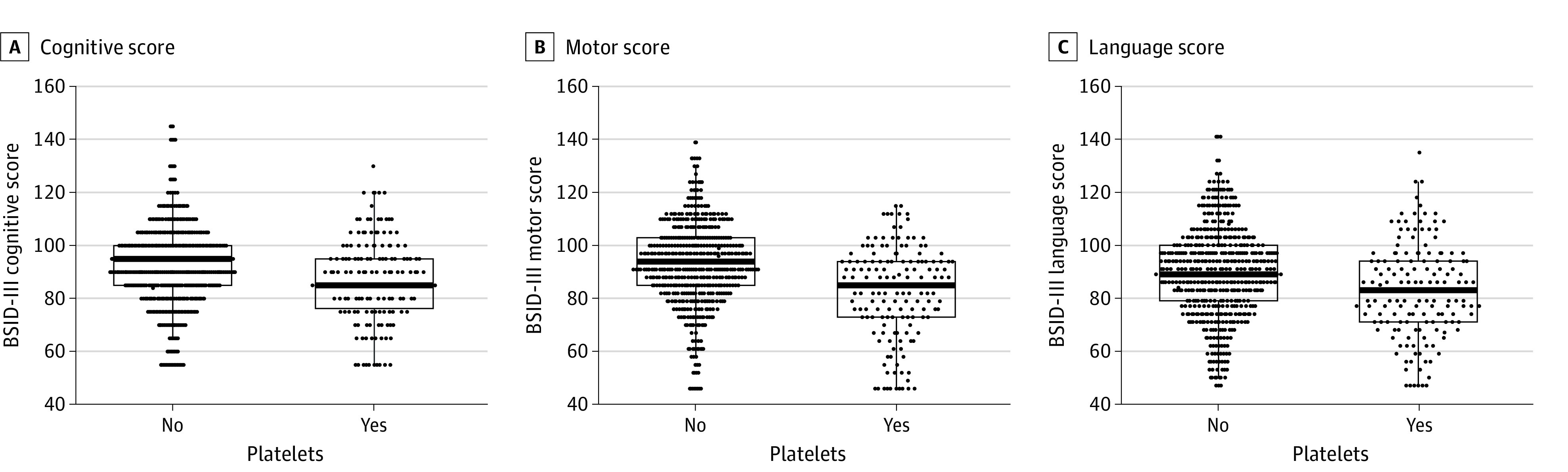
Cognitive, Motor and Language Scores by Platelet Exposure Dots represent the scores of individual infants on the Bayley Scales of Infant Development–Third Edition (BSID-III) cognitive (A), motor (B), and language scales (C).

The secondary outcome of death or moderate to severe NDI occurred in 68% of patients who received 1 or more platelet transfusion and 36% of those who did not receive any transfusion, with an AOR of 1.62 (95% CI, 0.83-3.17) (Table 2 and Figure 2). Moderate to severe NDI was present in 54% of patients who received 1 or more platelet transfusion and 33% of those who did not receive any transfusion, with an AOR of 1.02 (95% CI, 0.51-2.03). Infants who had received 1 or more transfusion also had lower mean motor scores at age 2 years’ corrected compared with those who had not received transfusions (Figure 3). Each additional platelet transfusion was associated with a 1.1-point decrease in mean motor score, but was not associated with cognitive or language scores (Table 2). In addition, since there is a possibility for erythropoietin treatment to impact megakaryopoiesis^26^ and thus endogenous platelet production, we evaluated the number of infants exposed to erythropoietin by treatment group and found that it was not different (49.7% vs 51.0%) (Table 1). The median number of platelet exposures was 2 (IQR, 1-4) in the erythropoietin treatment arm and 2 (IQR, 1-4) in the control arm (*P* = .60).

### Sensitivity Analyses

Five sensitivity analyses assessed the OR of death or NDI for any platelet exposure across 5 different models with AORs ranging from 2.43 to 3.46, all of which were statistically significant and directionally consistent with the primary analysis (eTable 1 in the Supplement). In addition, similar analysis in 4 models assessing the odds of death or NDI per each platelet exposure had AORs ranging from 1.22 to 1.24, all of which were statistically significant and directionally consistent with the primary analysis (eTable 2 in the Supplement).

## Discussion

In this observational cohort study, we found that exposure to platelet transfusion, after adjustment for confounding by indication, may be associated with a higher risk of death or severe NDI in children born extremely preterm. Platelet transfusion may also be associated with a higher risk of moderate to severe NDI in these children, and with lower motor scores on the BSID-III. These findings are consistent with the recently published 2-year outcomes from the PlaNet-2 Trial, which found a rate of death or significant NDI at a corrected age of 2 years of 50% in infants randomized to the higher platelet transfusion threshold of 50 × 10^3^/μL compared with 39% in infants randomized to the lower threshold of 25 × 10^3^/μL (OR, 1.54; 95% CI, 1.09-2.17; *P* = .02).^16^ In the PlaNet-2 Trial, 90% of infants in the high threshold group and 53% of infants in the low threshold group received at least 1 platelet transfusion. Thus, the larger magnitude of difference in NDI in our study may be due to the comparison of overall platelet exposure with no exposure, while the PlaNet-2 trial compared different transfusion thresholds resulting in variable levels of platelet transfusion exposure in both study arms.

The mechanisms underlying the possible worse neurodevelopmental outcomes associated with platelet transfusions remain unknown. Based on the findings of the PlaNeT-2 trial, the adverse long-term outcomes could be mediated by the higher incidence of major bleeding and bronchopulmonary dysplasia observed in infants randomized to the high compared with the low platelet transfusion threshold. However, it is unclear whether these short-term morbidities fully explain the worse long-term neurodevelopmental outcomes or whether transfused platelets have direct harmful effects on the developing brain. It is now recognized that, in addition to their important hemostatic functions, platelets are active participants in immune and inflammatory responses,^27,28^ and recent animal studies have reported that platelet transfusions can alter neonatal immune responses.^29,30^ It is therefore possible that transfused platelets may have direct proinflammatory effects on the preterm brain, although this remains speculative.

The 2-year follow-up study from the PlaNet-2 trial was the first to report an association between neonatal platelet transfusions and worse long-term neurodevelopmental outcomes and was particularly important given the randomized design of the original trial.^16^ However, outcomes in that study were ascertained using neurodevelopmental assessments based on multiple different types of follow-up, including parental questionnaires and clinician notes. Our study adds to the literature by reporting standardized assessment of 2-year neurodevelopmental outcomes through the use of the BSID-III assessments in all studied infants. This allowed us to investigate specific components of NDI, which suggest that platelet transfusion may have more adverse effects on motor function than cognitive or language function based on the specific BSID-III measures. We also evaluated the outcomes of exposure to increasing numbers of platelet transfusions with long term NDI.

### Limitations

Our study has several limitations. As an observational secondary analysis of a randomized clinical trial, we cannot be certain of the association between platelet transfusion exposure and 2-year outcomes despite our multiple approaches to address confounding. As expected, infants who received a platelet transfusion during their NICU admission were sicker compared with those who did not receive a transfusion. Although our use of propensity score methods to account for confounding by indication may have mitigated some of this treatment selection bias, it is likely we were unable to account for all potential confounders. In addition, there was a wide range of platelet transfusion frequencies between centers and it remains unclear whether the practices in these centers represented liberal or conservative approaches to platelet transfusion, given the lack of pretransfusion platelet count data. This finding is consistent with other studies that have shown the variability that exists between medical centers in the US regarding platelet transfusion approaches.^2^

## Conclusions

Together with the recent 2-year neurodevelopmental outcomes from the PlaNet-2 trial,^16^ our cohort study suggests that platelet transfusion exposure during NICU hospitalization may be associated with a higher risk of death and adverse 2-year neurodevelopmental outcomes in preterm infants. As with all observational studies, we cannot be certain of the association between platelet transfusion exposure and 2-year outcomes, despite multiple statistical approaches to address confounding. The mechanisms underlying the possible association remain unclear and further research is needed to elucidate them. While we await these answers, evidence-based platelet transfusion practices need to be implemented in this vulnerable population to reduce unnecessary platelet transfusions that might contribute to preventable harm.

## Notes

Supplement 1. **eFigure 1.** Reduction in Standardized Mean Difference After Stabilized Inverse Propensity Score Weighting **eFigure 2.** Distribution of Raw Propensity Scores by Platelet Exposure **eFigure 3.** Flow Diagram of Death and Neurodevelopmental Follow-up Rates for Transfused and Non-Transfused Infants Included in the Study **eFigure 4.** Variation in Platelet Exposure and Number of Infants Exposed by Study Site **eTable 1.** Sensitivity Analyses—Any Platelet Exposure **eTable 2.** Sensitivity Analyses—Number of Platelet Exposures

Supplement 2.
Data Sharing Statement
