# Supplementary material for: Platelet Transfusion and Death or Neurodevelopmental Impairment in Children Born Extremely Preterm
Source: JAMA Netw Open. 2024 Jan 23;7(1):e2352394. doi: 10.1001/jamanetworkopen.2023.52394 (PMC10807258; doi:10.1001/jamanetworkopen.2023.52394)
Supplement: Supplement 1. — eFigure 1. Reduction in Standardized Mean Difference After Stabilized Inverse Propensity Score Weighting eFigure 2. Distribution of Raw Propensity Scores by Platelet Exposure eFigure 3. Flow Diagram of Death and Neurodevelopmental Follow-up Rates for Transfused and Non-Transfused Infants Included in the Study eFigure 4. Variation in Platelet Exposure and Number of Infants Exposed by Study Site eTable 1. Sensitivity Analyses—Any Platelet Exposure eTable 2. Sensitivity Analyses—Number of Platelet Exposures [file jamanetwopen-e2352394-s001.pdf]

Supplementary Online Content

Davenport PE, Wood TR, Heagerty PJ, Sola-Visner MC, Juul SE, Patel RM. Platelet transfusion and death or neurodevelopmental impairment in children born extremely preterm. *JAMA Netw Open*. 2024;7(1):e2352394.
doi:10.1001/jamanetworkopen.2023.52394

eFigure 1. Reduction in Standardized Mean Difference After Stabilized Inverse Propensity Score Weighting

eFigure 2. Distribution of Raw Propensity Scores by Platelet Exposure

eFigure 3. Flow Diagram of Death and Neurodevelopmental Follow-up Rates for Transfused and Non-Transfused Infants Included in the Study

eFigure 4. Variation in Platelet Exposure and Number of Infants Exposed by Study Site

eTable 1. Sensitivity Analyses—Any Platelet Exposure

eTable 2. Sensitivity Analyses—Number of Platelet Exposures

This supplementary material has been provided by the authors to give readers additional information about their work.

© 2024 Davenport PE et al. *JAMA Network Open.*

eFigure 1. Reduction in Standardized Mean Difference after Stabilized Inverse Propensity Score Weighting.

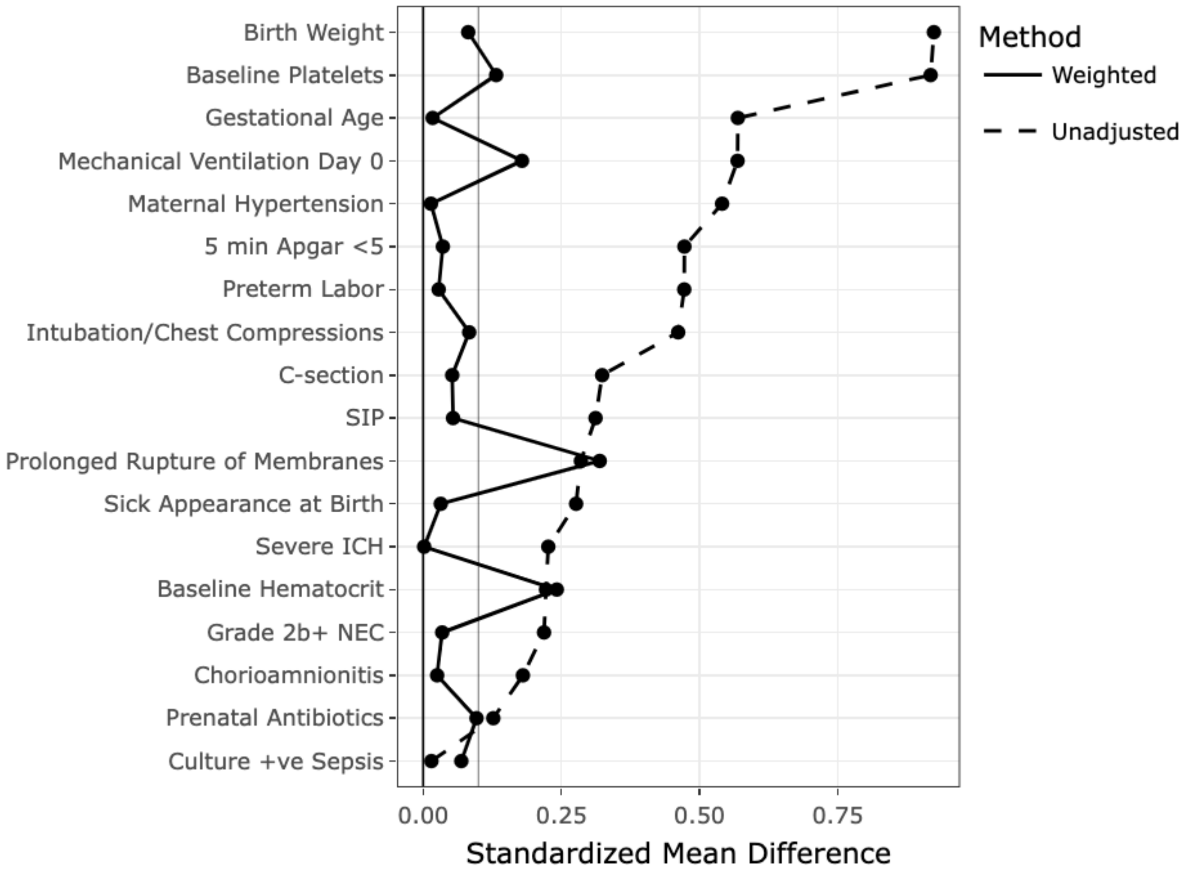

Abbreviations: SIP, spontaneous intestinal perforation; ICH, intracranial/intraventricular hemorrhage; NEC, necrotizing enterocolitis.
All events are those occurring prior to first platelet exposure.

© 2024 Davenport PE et al. *JAMA Network Open.*

eFigure 2. Distribution of Raw Propensity Scores by Platelet Exposure.

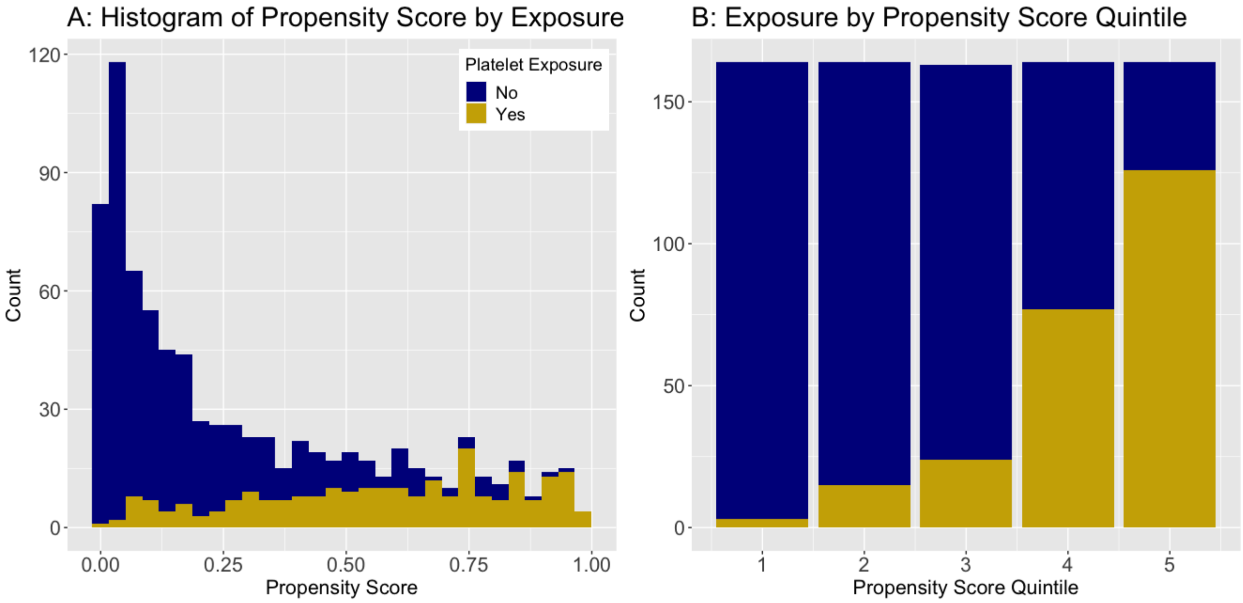

© 2024 Davenport PE et al. *JAMA Network Open.*

eFigure 3. Flow Diagram of Death and Neurodevelopmental Follow-up Rates for Transfused and Non-Transfused Infants Included in the Study.

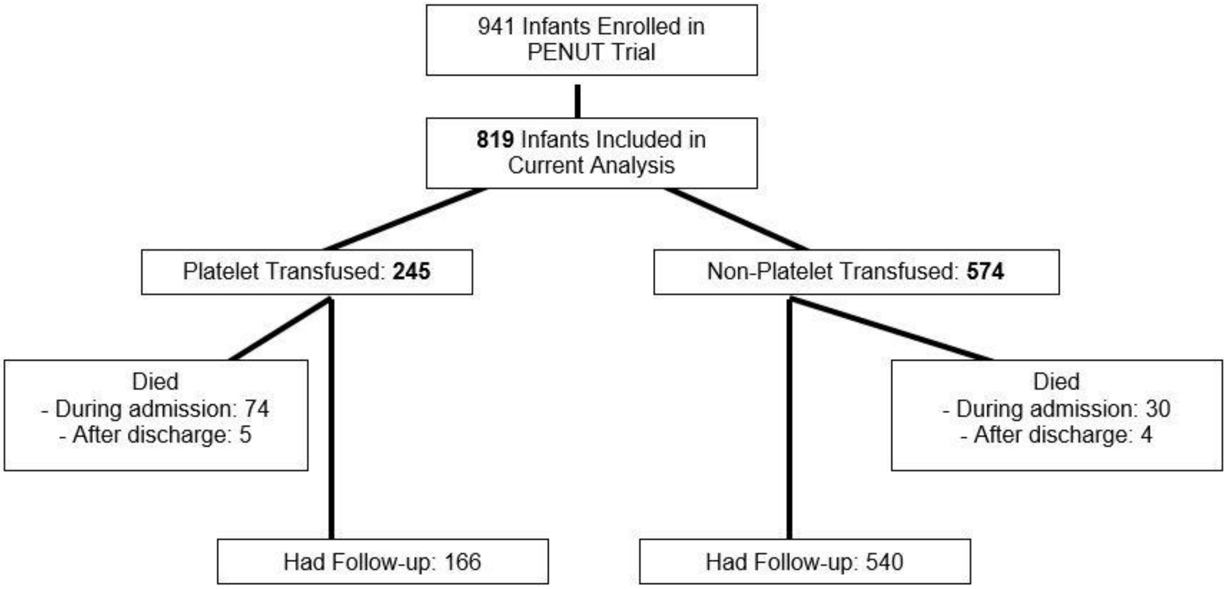

© 2024 Davenport PE et al. *JAMA Network Open.*

eFigure 4. Variation in Platelet Exposure and Number of Infants Exposed by Study Site

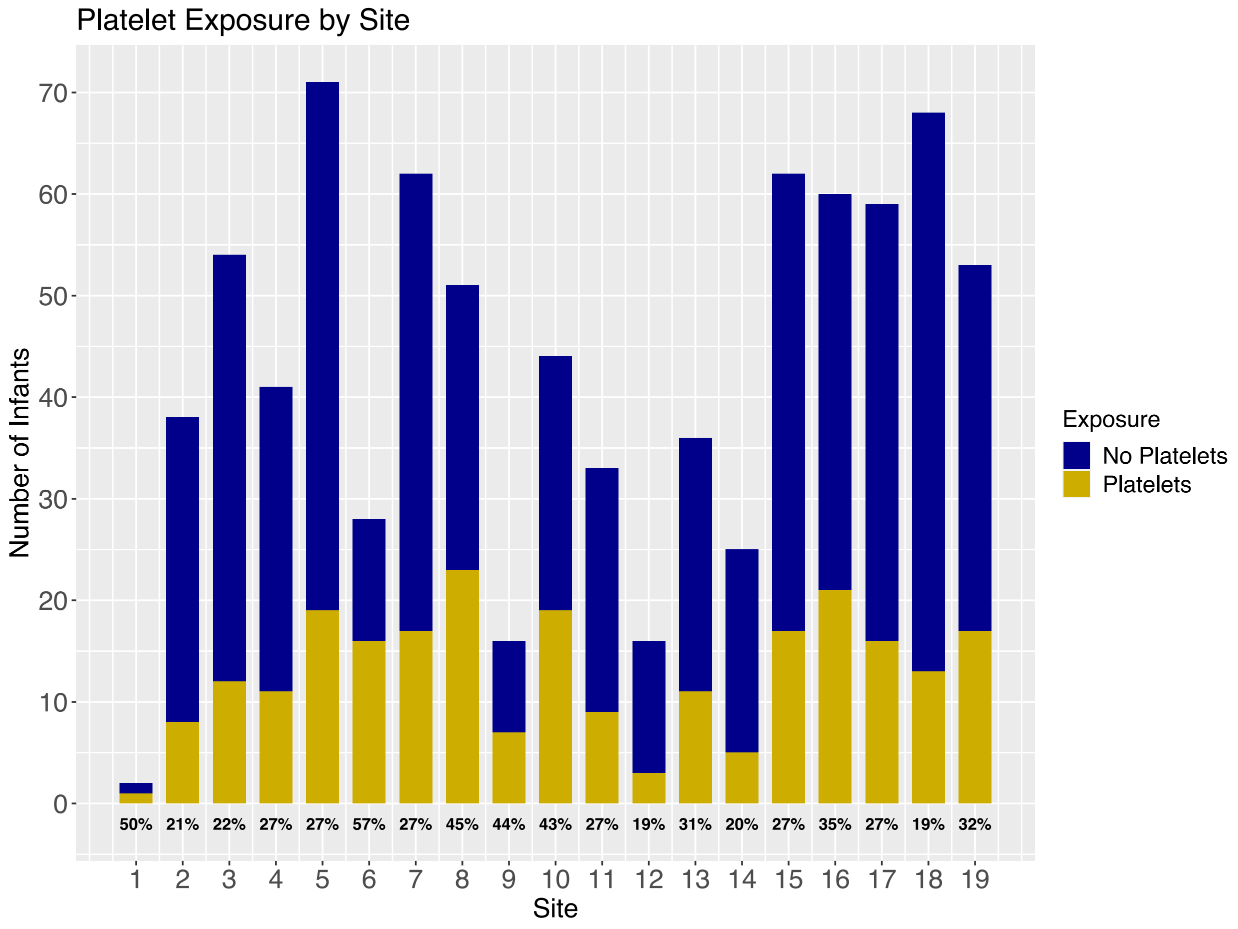

© 2024 Davenport PE et al. *JAMA Network Open.*

eTable 1. Sensitivity Analyses – Any Platelet Exposure

**Outcomes**	**Adjusted OR or Adjusted Mean Difference (95% CI) for Any Platelet Exposure vs. No Exposure**				
Primary Outcome	**Sensitivity 1**	**Sensitivity 2**	**Sensitivity 3**	**Sensitivity 4**	**Sensitivity 5**
Primary outcome (death/severe NDI)	**2.43 (1.28, 4.59)**	**2.62 (1.74, 4.14)**	**3.46 (2.30, 5.23)**	**3.20 (2.52, 7.38)**	**2.90 (1.89, 4.46)**
Components of Outcome					
Death	**3.79 (1.84, 7.82)**	**3.91 (2.21, 6.90)**	**4.54 (2.72, 7.56)**	**4.12 (2.29, 7.44)**	**4.06 (2.31, 7.12)**
Severe NDI (survivors)	0.98 (0.45, 2.13)	1.43 (0.77, 2.65)	**1.98 (1.11, 3.52)**	1.65 (0.87, 3.13)	**1.89 (0.997, 3.57)**
Secondary Outcomes *(binary)*					
Death or Moderate-Severe NDI	1.65 (0.92, 2.97)	**2.16 (1.47, 3.17)**	**2.72 (1.87, 3.94)**	**2.39 (1.56, 3.66)**	**2.14 (1.46, 3.13)**
Moderate-Severe NDI (survivors)	1.04 (0.56, 1.93)	1.45 (0.93, 2.26)	**1.80 (1.19, 2.74)**	1.51 (0.94, 2.43)	1.50 (0.96, 2.33)
Secondary Outcomes *(continuous)*					
BSID-III Cognitive	3.1 (-3.4, 9.6)	-0.9 {-4.3, 2.5)	-2.6 (-5.8, -0.6)	-2.2 (-5.6, 1.3)	-2.0 (-5.1, 1.1)
BSID-III Motor	**-4.4 (-8.1, -0.7)**	**-5.5 (-9.3, -1.7)**	**-6.6 (-10.1, -3.1)**	**-6.3 (-10.1, 2.5)**	**-5.8 (-9.3, -2.4)**
BSID-III Language	-0.5 (-6.7, 5.8)	-3.0 {-7.0, -1.0)	**-3.6 (-7.2, -0.02)**	-2.1 (-6.4, 2.2)	**-3.8 (-7.5, -0.1)**

Bolded point estimates are those whose 95% CIs do not cross 1 (aOR) or 0 (BSID-III subscales)
Sensitivity 1 - Main models with baseline platelet level, baseline hematocrit level, prolonged rupture of membranes , and mechanical ventilation at enrollment included as additional covariates
Sensitivity 2 - Unweighted GEE adjusting for propensity score quintiles
Sensitivity 3 - 1:1 propensity score matched GEE model with clustering by matched pairings
Sensitivity 4 - Weighted GEE models after optimal trimming of outlying propensity scores
Sensitivity 5 - Full regression models with all propensity score variables used as covariates

© 2024 Davenport PE et al. *JAMA Network Open.*

eTable 2. Sensitivity Analyses – Number of Platelet Exposures

**Outcomes**	**Adjusted OR or Adjusted Mean Difference (95% CI) per Each Additional Platelet Transfusion (up to 20)**			
Primary Outcome	Sensitivity 1	Sensitivity 2	Sensitivity 3	Sensitivity 4
Primary outcome (death/severe NDI)	**1.24 (1.05, 1.46)**	**1.22 (1.12, 1.33)**	**1.24 (1.11, 1.39)**	**1.23 (1.13, 1.34)**
Components of Outcome				
Death	**1.21 (1.06, 1.40)**	**1.14 (1.07, 1.21)**	**1.16 (1.06, 1.28)**	**1.14 (1.07, 1.22)**
Severe NDI (survivors)	1.11 (0.999, 1.24)	**1.19 (1.07, 1.31)**	**1.17 (1.03, 1.32)**	**1.22 (1.11, 1.33)**
Secondary Outcomes *(binary)*				
Death or Moderate-Severe NDI	1.15 (0.99, 1.34)	**1.15 (1.05, 1.27)**	**1.18 (1.05, 1.33)**	**1.16 (1.05, 1.29)**
Moderate-Severe NDI (survivors)	1.05 (0.93, 1.19)	1.09 (0.99, 1.19)	1.09 (0.97, 1.21)	**1.10 (1.003, 1.20)**
Secondary Outcomes *(continuous)*				
BSID-III Cognitive	-0.2 (-1.3, 1.0)	-0.5 (-1.3, 0.2)	-0.5 (-1.3, 0.4)	-0.6 (-1.2, 0.1)
BSID-III Motor	-1.0 (-2.1, 0.1)	**-1.1 (-1.8, -0.3)**	**-1.3 (-2.3, -0.3)**	**-1.3 (-2.0, -0.6)**
BSID-III Language	-0.6 (-1.7, 0.5)	-0.6 (-1.4, 0.1)	-0.3 (-1.2, -0.5)	**-0.9 (-1.5, -0.2)**

Bolded point etimates are those whose 95% CIs do not cross 1 (aOR) or 0 (BSID-III subscales)
Sensitivity 1 - Main models with baseline platelet level, baseline hematocrit level, prolonged rupture of membranes, and mechanical ventilation at enrolment as additional covariates
Sensitivity 2 - 1:1 propensity score matched GEE model with clustering by matched pairings
Sensitivity 3 - Weighted GEE models after optimal trimming of outlying propensity scores
Sensitivity 4 - Full regression models with all propensity score variables used as covariates

© 2024 Davenport PE et al. *JAMA Network Open.*
