# Supplementary material for: Platelet Transfusion and Death or Neurodevelopmental Impairment in Children Born Extremely Preterm
Source: JAMA Netw Open. 2024 Jan 23;7(1):e2352394. doi: 10.1001/jamanetworkopen.2023.52394 (PMC10807258; doi:10.1001/jamanetworkopen.2023.52394)
Supplement: Supplement 2. — Data Sharing Statement [file jamanetwopen-e2352394-s002.pdf]

Data Sharing Statement

Davenport. Platelet Transfusion and Death or Neurodevelopmental Impairment in Children Born Extremely Preterm. *JAMA Netw Open*. Published January 23, 2024. doi:10.1001/jamanetworkopen.2023.52394

Data
Data available: Yes
Data types: Deidentified participant data
How to access data: Anyone can request open access to the PENUT data set by following the instructions on the NINDS page: https://www.ninds.nih.gov/current-research/research-funded-ninds/clinical-research/archived-clinical-research-datasets
When available: With publication

Supporting Documents
Document types: None

Additional Information
Who can access the data: Anyone can request open access to the PENUT data set by following the instructions on the NINDS page: https://www.ninds.nih.gov/current-research/research-funded-ninds/clinical-research/archived-clinical-research-datasets
Types of analyses: Anyone can request open access to the PENUT data set by following the instructions on the NINDS page: https://www.ninds.nih.gov/current-research/research-funded-ninds/clinical-research/archived-clinical-research-datasets
Mechanisms of data availability: Anyone can request open access to the PENUT data set by following the instructions on the NINDS page: https://www.ninds.nih.gov/current-research/research-funded-ninds/clinical-research/archived-clinical-research-datasets
